# A Note on the Mixing Factor of Polar Codes

**DOI:** 10.3390/e25111498

**Published:** 2023-10-30

**Authors:** Keer Wei, Xiaoyu Jin, Weihua Yang

**Affiliations:** School of Mathematics, Taiyuan University of Technology, Taiyuan 030024, China; weikeerco@163.com (K.W.); jinxiaoyu1620@163.com (X.J.)

**Keywords:** decreasing monomial codes, mixing factor, polar codes

## Abstract

Over binary-input memoryless symmetric (BMS) channels, the performance of polar codes under successive cancellation list (SCL) decoding can approach maximum likelihood (ML) algorithm when the list size *L* is greater than or equal to $2^{MF}$, where MF, known as mixing factor of code, represents the number of information bits before the last frozen bit. Recently, Yao et al. showed the upper bound of the mixing factor of decreasing monomial codes with length $n=2^{m}$ and rate $R\leq \frac{1}{2}$ when *m* is an odd number; moreover, this bound is reachable. Herein, we obtain an achievable upper bound in the case of an even number. Further, we propose a new decoding hard-decision rule beyond the last frozen bit of polar codes under BMS channels.

## 1. Introduction

Polar code [1], a channel coding scheme theoretically able to reach the Shannon limit, is proposed by Arıkan based on polarization phenomenon. In the encoding process, polar codes have a clear and explicit structure: the codeword set is a linear space spanned by given rows of the Kronecker power of a special second-order polarization matrix. Meanwhile, Arıkan adopted a successive cancellation (SC) decoding scheme [1] with low complexity. In practical applications, an efficient decoding algorithm is one of the decisive factors for judging the ability of a coding system. SC decoding of polar codes has low error probability in long code length but poor performance in finite code length. Therefore, Tal and Vardy proposed the SCL decoding algorithm [2] to attain better performance. From the perspective of improving polar codes with finite length, a series of concatenation codes are utilized to ameliorate the code spectrum and approach the ML bound, such as cyclic redundancy check (CRC) polar codes [2], parity check (PC) polar codes [3], and polarization-adjusted convolutional (PAC) codes [4].

At present, most researchers focus on SCL decoding with good performance, since its error probability can coincide with ML decoding when the list size *L* tends to infinity. In [5], it is proved theoretically that under binary erasure channels (BEC), the SCL decoding of $\left(n,k\right)$ polar codes with $L=2^{MF}$ can be close to optimal maximum a posteriori (MAP) performance, and numerical experiments presented that the above conclusion holds for general channels such as binary-input additive white Gaussian noise (BI-AWGN) channels. Fazeli et al. further proved that the SCL decoding of polar codes with length *n* can achieve the performance of ML algorithm under BMS channels when *L* is greater than or equal to $2^{MF}$ and designed a hybrid decoder between SCL and the nearest coset algorithm; hence, the upper bound of ML decoding complexity for polar codes is $O\left(2^{MF}nlogn\right)$ [6]. Both simulation experiments in [6,7] showed that if a Reed–Muller (RM) code and a polar code have the same length and dimension, the MF value corresponding to the RM code is larger; this implies, compared with general polar codes, that the PAC codes with RM rate profiling need a larger value *L* to close ML performance under SCL decoding. Aiming to obtain the entire weight distribution of polar codes, [8] used a recursive algorithm to calculate the weight enumeration function (WEF) of a polar coset with quadratic polynomial complexity, since polar codes can be regarded as the union of some polar cosets. And Yao et al. also showed the upper bound of the mixing factor of decreasing monomial codes with length $n=2^{m}$ and dimension $k\leq \frac{n}{2}$ when *m* is an odd number, so as to limit the complexity of the total algorithm bounded loosely by $O\left(2^{MF}n^{2}\right)$ in [8].

In this paper, we first show the upper bound of the mixing factor of decreasing monomial codes with code length $n=2^{m}$ and code rate $R\leq \frac{1}{2}$, when *m* is an even number. Meanwhile, we certify that this bound is reachable, but the achievable condition is distinct under different code lengths. Further, we propose a new decoding hard-decision rule with respect to the Hamming distance between cosets and the given vector beyond the last frozen bit of polar codes over BMS channels.

## 2. Preliminary

In this part, we review some basic knowledge about polar codes and decreasing monomial codes.

### 2.1. Polar Codes

Let the (n,k) code represent a polar code with length *n*, dimension *k* and rate $R=\frac{k}{n}$; the information set used to transmit messages and the frozen set used to convey fixed bits are, respectively, denoted by I and F, where $I,F\subseteq \left\{0,1,\cdots ,n-1\right\}$. The information set I contains indices of *k* reliable subchannels $W_{n}^{\left(i\right)}\left(i\in \left\{0,1,\cdots ,n-1\right\}\right)$ gained by density evolution (DE) [9], Tal–Vardy algorithm [10], Gaussian approximation (GA) [11], polarization weight (PW) construction [12] and so on. Here, we consider the frozen bit to be zero. The codeword set of a polar code is generated by the product of information sequence and Kronecker power of $G_{2}=\left[\begin{array}{cc} 1 & 0\\ 1 & 1 \end{array}\right]$, i.e., $x_{0}^{n-1}=u_{0}^{n-1}G_{2}^{⊗m}$, where $n=2^{m}$, $a_{i}^{j}=\left(a_{i},a_{i+1},\cdots ,a_{j}\right)\in {\left\{0,1\right\}}^{j-i+1}$ and ⊗ is the Kronecker product. RM codes with length $n=2^{m}$ and order r≤m, denoted by RM(r,m), are also generated by $G_{2}^{⊗m}$. Compared with polar codes constructed on subchannels reliability, the information set selection method of RM codes relies on the row weight of generated matrix $G_{2}^{⊗m}$, which is related with the binary expression of the row index.

SC decoding is a classic decoding scheme for polar codes with length $n=2^{m}$, which uses *n* received signals $y_{0}^{n-1}$ and *i* decoded bits ${\hat{u}}_{0}^{i-1}$ to estimate the *i*-th bit $u_{i}$. The following SC hard-decision rule is based on subchannel transition probabilities $W_{n}^{\left(i\right)}\left(y_{0}^{n-1},{\hat{u}}_{0}^{i-1}|u_{i}\right),i\in \left\{0,1,\cdots ,n-1\right\}$.

(1) $${\hat{u}}_{i}=\left\{\begin{array}{cc} u_{i}, & i\in F\\ 0, & i\in I\;and\;\frac{W_{n}^{\left(i\right)}\left(y_{0}^{n-1},{\hat{u}}_{0}^{i-1}|u_{i}=0\right)}{W_{n}^{\left(i\right)}\left(y_{0}^{n-1},{\hat{u}}_{0}^{i-1}|u_{i}=1\right)}\geq 1\\ 1, & i\in I\;and\;\frac{W_{n}^{\left(i\right)}\left(y_{0}^{n-1},{\hat{u}}_{0}^{i-1}|u_{i}=0\right)}{W_{n}^{\left(i\right)}\left(y_{0}^{n-1},{\hat{u}}_{0}^{i-1}|u_{i}=1\right)}<1 \end{array}\right.$$

The SCL algorithm is an aggregation of SC decoders, which can preserve *L* decoding results with the smallest *L* path metric values and exceed ML performance combined with CRC-Aided [13]. In addition, belief propagation (BP) [14], successive cancellation stack (SCS) [15], etc., decoding schemes also improve the performance of polar codes to a certain extent.

### 2.2. Decreasing Monomial Codes

Let $M_{m}=\left\{x_{0}^{a_{0}}x_{1}^{a_{1}}\cdots x_{m-1}^{a_{m-1}}|a_{i}\in \left\{0,1\right\},i\in \left\{0,1,\cdots ,m-1\right\}\right\}$ denote a monomial set with *m* variables and ind(f) represent a set containing all variables that appear in monomial $f\in M_{m}$. Due to the particularity of structure of $G_{2}^{⊗m}$, each row $g_{i}$ of $G_{2}^{⊗m}={\left[g_{0}^{T},g_{1}^{T},\cdots ,g_{2^{m}-1}^{T}\right]}^{T}$ can be represented by a specific monomial, where *T* denotes the transpose. Let $bin\left(i\right)=i_{m-1}i_{m-2}\cdots i_{1}i_{0}$ be the binary expansion of $i\in \left\{0,1,\cdots ,2^{m}-1\right\}$, where $i=\sum_{k=0}^{m-1}i_{k}2^{k}$. Then, each row index *i* of $G_{2}^{⊗m}$ uniquely corresponds to a monomial *f*; for simplicity, we use $i_{f}$ to signify row index and monomial, i.e., $f=\;x_{0}^{1-i_{f,0}}\cdots x_{m-1}^{1-i_{f,m-1}}$, where $bin\left(i_{f}\right)=i_{f,m-1}\cdots i_{f,1}i_{f,0}$. The following are the row index and corresponding monomial expression of $G_{2}^{⊗3}$.

(2) $$\begin{array}{c} \begin{array}{c} \begin{array}{c} \begin{array}{c} \begin{array}{c} 0_{x_{0}x_{1}x_{2}}\\ 1_{x_{1}x_{2}} \end{array}\\ 2_{x_{0}x_{2}} \end{array}\\ 3_{x_{2}} \end{array}\\ 4_{x_{0}x_{1}} \end{array}\\ 5_{x_{1}}\\ 6_{x_{0}}\\ 7_{1} \end{array}\left[\begin{array}{cccc} \begin{array}{cc} 1 & 0\\ 1 & 1 \end{array} & \begin{array}{cc} 0 & 0\\ 0 & 0 \end{array} & \begin{array}{cc} 0 & 0\\ 0 & 0 \end{array} & \begin{array}{cc} 0 & 0\\ 0 & 0 \end{array}\\ \begin{array}{cc} 1 & 0\\ 1 & 1 \end{array} & \begin{array}{cc} 1 & 0\\ 1 & 1 \end{array} & \begin{array}{cc} 0 & 0\\ 0 & 0 \end{array} & \begin{array}{cc} 0 & 0\\ 0 & 0 \end{array}\\ \begin{array}{cc} 1 & 0\\ 1 & 1 \end{array} & \begin{array}{cc} 0 & 0\\ 0 & 0 \end{array} & \begin{array}{cc} 1 & 0\\ 1 & 1 \end{array} & \begin{array}{cc} 0 & 0\\ 0 & 0 \end{array}\\ \begin{array}{cc} 1 & 0\\ 1 & 1 \end{array} & \begin{array}{cc} 1 & 0\\ 1 & 1 \end{array} & \begin{array}{cc} 1 & 0\\ 1 & 1 \end{array} & \begin{array}{cc} 1 & 0\\ 1 & 1 \end{array} \end{array}\right]\begin{array}{c} \begin{array}{c} \begin{array}{c} \begin{array}{c} \begin{array}{c} x_{0}x_{1}x_{2}\\ x_{1}x_{2} \end{array}\\ x_{0}x_{2} \end{array}\\ x_{2} \end{array}\\ x_{0}x_{1} \end{array}\\ x_{1}\\ x_{0}\\ 1 \end{array}$$

It is easy to see that both polar codes and RM codes are spanned by monomials; hence, we call the code of this form a monomial code. Further, Bardet et al. in [16] first defined partial order ≼, in addition to proposing the concept of decreasing monomial codes and their main properties and checking that polar codes and RM codes belong to decreasing monomial codes.

**Definition 1**  (Decreasing Polar Codes) [16])**.** *A set $I\subseteq M_{m}$ is decreasing if and only if (f∈I and g≼f) implies g∈I. When $I\subseteq M_{m}$ is a decreasing set, then C(I) is called a decreasing monomial code.*

In this paper, we directly use the symbols, definitions and conclusions from [16]. Note, we do not distinguish whether the information set I contains row indices or monomials.

## 3. The Upper Bound of the Mixing Factor

In [6], the concept of mixing factor is proposed based on the index of the last frozen bit. Next, as represented by Theorem 1, Yao et al. proved the upper bound of the mixing factor of decreasing monomial codes with length $2^{m}$ and dimension $k\leq 2^{m-1}$ achievable when *m* is an odd number; however, they only speculated on the case of even numbers *m* [8]. Therefore, we mainly prove Theorem 2 to make Theorem 1 complete in this section.

**Definition 2**  (Mixing Factor)**.** *Let C be a decreasing monomial code with length n and information set I and $\tau \left(C\right)$ represent the index of the last frozen bit; then, we call the number of information bits before $\tau \left(C\right)$ as the mixing factor of C, denoted by MF(C). Obviously, $\tau \left(C\right)$ and MF(C) satisfy the following equation:*

(3) $$MF\left(C\right)=\tau \left(C\right)-\left(n-\left|I\right|\right)+1.$$

**Theorem 1**  ([8])**.** *Let C be an (n,k) polar code with length $n=2^{m}$, m=2t+1 and dimension $k\leq \frac{n}{2}$, then*

(4) $$MF\left(C\right)\leq 2^{2t}-2^{t+1}+1.$$

*Moreover, the equality holds only when C is the self-dual Reed–Muller code.*

**Theorem 2.**  *Let C be a decreasing monomial code with length $n=2^{m}$, m=2t and rate $R\leq \frac{1}{2}$, then*

(5) $$MF\left(C\right)\leq 2^{2t-1}-2^{t+1}+2.$$

*Moreover, the equality holds for t≥3 if and only if C is a subcode of RM(t,2t) code with the information set $I=\left\{f\in M_{m}|deg\left(f\right)\leq t-1\right\}\cup \left\{g\in M_{m}|deg\left(g\right)=t,x_{0}\in ind\left(g\right)\right\}$. In the case of t=1 and t=2, the equality holds if and only if C is an RM(t−1,2t) code or C is a subcode of RM(t,2t) code with the information set $I=\left\{f\in M_{m}|deg\left(f\right)\leq t-1\right\}\cup \left\{g\in M_{m}|deg\left(g\right)=t,\right.$$\left.x_{0}\in ind\left(g\right)\right\}$.*

As shown in Table 1, we obtain some of the largest MF values according to inequality (5). In the following, we analyze certain special cases with $t\in \left\{1,2,3,4,5\right\}$.

If t=1 and t=2, we consider the decreasing monomial codes with $\tau \left(C\right)\geq 2^{2t-1}-1$, since the code rate $R\leq \frac{1}{2}$. By exhaustive research, we can obtain the conclusions of Theorem 2. For example, let t=2 and τ(C)=9, then the monomial corresponding to row index 9 is $f=x_{1}x_{2}$. Aiming to count the number of information bits above τ(C), we initiate the enumeration of frozen bits, except for *f*; there are 7 monomials $g_{i}$ satisfying $f≼g_{i}$, where $i\in \left\{1,2,\cdots ,7\right\}$, respectively, corresponding to index $0_{x_{0}x_{1}x_{2}x_{3}}$, $1_{x_{1}x_{2}x_{3}}$, $2_{x_{0}x_{2}x_{3}}$, $3_{x_{2}x_{3}}$, $4_{x_{0}x_{1}x_{3}}$, $5_{x_{1}x_{3}}$, $8_{x_{0}x_{1}x_{2}}$. Therefore, there are at least 8 frozen bits due to the property of decreasing monomial codes, and then MF(C)≤2 from (3); moreover, MF(C)=2 when C is exactly a subcode of RM(2,4) code and the information set includes $6_{x_{0}x_{3}}$, $7_{x_{3}}$, $10_{x_{0}x_{2}}$, $11_{x_{2}}$, $12_{x_{0}x_{1}}$, $13_{x_{1}}$, $14_{x_{0}}$, $15_{1}$. Besides, if the decreasing monomial code is RM(1,4) code, then τ(C)=12 and MF(C)=2.

If 3≤t≤5, we also consider cases under $\tau \left(C\right)\geq 2^{2t-1}-1$ to obtain the conclusion of Theorem 2 by exhaustive research. Compared with the previous case on $t\in \left\{1,2\right\}$, there is only one necessary and sufficient condition for reaching the largest mixing factor value in this case.

In the following, we prove the upper bound of the mixing factor of decreasing monomial codes achievable by Lemmas 1 and 5 when t≥5.

**Remark 1.**  *By observing Table 2, in which we omit the writing of the bottom right corner marker f in $i_{f}$, the degree of monomials under monomial h is no larger than t, especially if the degree is exactly t, then the monomial includes variable $x_{0}$.*

**Lemma 1.**  *$n=2^{m}$, m=2t, let C be a subcode of RM(t,2t) code with information set $I=\left\{f\in M_{m}|deg\left(f\right)\leq t-1\right\}\cup \left\{g\in M_{m}|deg\left(g\right)=t,x_{0}\in ind\left(g\right)\right\}$, then $MF\left(C\right)=2^{2t-1}-2^{t+1}+2$.*

**Proof.**  First, we enumerate information set I:

(6) $$\begin{array}{cc} \left|I\right|\; & =\left|\left\{f\in M_{m}|deg\left(f\right)\leq t-1\right\}\right|+\left|\left\{g\in M_{m}|deg\left(g\right)=t,x_{0}\in ind\left(g\right)\right\}\right|\\ \; & =\sum_{k=0}^{t-1}C_{2t}^{k}+\frac{1}{2}C_{2t}^{t}\\ \; & =2^{2t-1}. \end{array}$$

Thus, the dimension of code C is $\frac{n}{2}$. Next, according to Table 2, for any $p\in M_{m}$ satisfying $i_{p}\geq i_{h}$, we have 0≤deg(p)≤t and $x_{0}\in ind\left(p\right)$ when deg(p)=t, then p∈I. Therefore, $\tau \left(C\right)=i_{h}$ and $MF\left(C\right)=2^{2t-1}-2^{t+1}+2$ from (3). □

**Lemma 2.**  *$n=2^{m}$, m=2t, let C be a decreasing monomial code with length n and frozen set F. If $\tau \left(C\right)\geq i_{h}$, then $i_{h}\in F$.*

**Proof.**  By observation of Table 2, for any $p\in M_{m}$ satisfying $i_{p}\geq i_{h}$, we have p⪯h. Thus, let $i_{p^{\prime }}=\tau \left(C\right)\geq i_{h}$, then $p^{\prime }⪯h$, that is $i_{h}\in F$ because of the property of decreasing monomial codes. □

**Lemma 3.**  *$n=2^{m}$, m=2t, let C be a decreasing monomial code with length n and frozen set F. If $\tau \left(C\right)\geq i_{h^{\prime }}$, then $i_{h^{\prime }}\in F$.*

**Proof.**  Similarly to the proof of Lemma 2, $p⪯h^{\prime }$ holds for any $p\in M_{m}$ satisfying $i_{p}\geq i_{h^{\prime }}$. Let $i_{p^{\prime \prime }}=\tau \left(C\right)\geq i_{h^{\prime }}$, then $p^{\prime \prime }⪯h^{\prime }$, that is $i_{h^{\prime }}\in F$ because of the property of decreasing monomial codes. □

**Lemma 4.**  *Let C be a decreasing monomial code with length $n=2^{m}$, m=2t, rate $R\leq \frac{1}{2}$ and frozen set F. If $MF\left(C\right)\geq 2^{2t-1}-2^{t+1}+2$ and $\tau \left(C\right)=i_{h}$, then C is a subcode of RM(t,2t) code with information set $I=\left\{f\in M_{m}|deg\left(f\right)\leq t-1\right\}\cup \left\{g\in M_{m}|deg\left(g\right)=t,x_{0}\in ind\left(g\right)\right\}$.*

**Proof.**  Obviously, for any $p\in M_{m}$ satisfying deg(p)≥t+1, we have h⪯p; thus, p∈F because of the decreasing code C. This implies C is a subcode of RM(t,2t) code and the dimension of this subcode is no larger than $\sum_{k=0}^{t}C_{2t}^{k}$.

On the other hand, for any $p\in \left\{f|deg(f)\geq t+1\right\}\cup \left\{g|deg\left(g\right)=t,x_{0}\notin ind\left(g\right)\right\}$, we have h⪯p, then p∈F. Thus, we know the size of the frozen set of code C:

(7) $$\left|F\right|\geq C_{2t-1}^{t}+\sum_{k=t+1}^{2t}C_{2t}^{k}=2^{2t-1},$$

that is, $\left|I\right|\leq \frac{n}{2}$. By applying 3, we obtain

(8) $$\left|I\right|=MF\left(C\right)-\tau \left(C\right)+\left(n-1\right)\geq \frac{n}{2}.$$

This implies $\left|I\right|=\frac{n}{2}$ from (7) and (8); hence, we deduce that information set of code C is $\left\{f\in M_{m}|deg\left(f\right)\leq t-1\right\}\cup \left\{g\in M_{m}|deg\left(g\right)=t,x_{0}\in ind\left(g\right)\right\}$. □

**Lemma 5.**  *t≥5, let C be a decreasing monomial code with length $n=2^{m}$, m=2t, rate $R\leq \frac{1}{2}$ and frozen set F. If $MF\left(C\right)\geq 2^{2t-1}-2^{t+1}+2$, then C is only a subcode of RM(t,2t) code with the information set $I=\left\{f\in M_{m}|deg\left(f\right)\leq t-1\right\}\cup \left\{g\in M_{m}|deg\left(g\right)=t,x_{0}\in ind\left(g\right)\right\}$.*

**Proof.**  First, by applying (3), we have $\tau \left(C\right)\geq 2^{2t}-2^{t+1}+1=i_{h}$.

Next, we are ready to find the contradiction to verify $\tau \left(C\right)=i_{h}$, then suppose $\tau \left(C\right)>i_{h}$. From Lemmas 2 and 3, we have $i_{h},i_{h^{\prime }}\in F$, and then enumerate the elements in the frozen set F. According to $i_{h}\in F$, for any $p\in \left\{f\in M_{m}|deg\left(f\right)\geq t+1\right\}\cup \left\{g\in M_{m}|deg\left(g\right)=t,x_{0}\notin ind\left(g\right)\right\}$, then h⪯p, $i_{p}\in F$, this implies

(9) $$\left|F\right|\geq \sum_{k=t+1}^{2t}C_{2t}^{k}+C_{2t-1}^{t}.$$

Then, consider $i_{h^{\prime }}\in F$, for any $p\in \left\{f\in M_{m}|deg\left(f\right)=t,x_{0}\in ind\left(f\right),x_{1}\notin ind\left(f\right)\right\}$, then $h^{\prime }⪯p$. Hence, the number of monomials satisfying the above conditions is $C_{2t-2}^{t-1}$.

Therefore, we have

(10) $$\left|F\right|\geq \sum_{k=t+1}^{2t}C_{2t}^{k}+C_{2t-1}^{t}+C_{2t-2}^{t-1}=2^{2t-1}+C_{2t-2}^{t-1},$$

then

(11) $$\left|I\right|=n-\left|F\right|\leq 2^{2t-1}-C_{2t-2}^{t-1}.$$

If t≥5, this is a contradiction for

(12) $$\left|I\right|<2^{2t-1}-2^{t+1}+2\leq MF\left(C\right),$$

we have $\tau \left(C\right)=i_{h}$. From Lemma 4, we can finish the proof. □

Finally, due to Lemmas 1 and 5, we can easily conclude Theorem 2. Therefore, the complexity bound of the ML decoding algorithm in polar codes can obviously be obtained.

Next, we adopt the PW method [12] to gain the information sets of (n,k) polar codes with $k=\frac{n}{4}$ and $k=\frac{n}{2}$, respectively, then compare their mixing factors with the largest MF values from (5) under distinct lengths, as shown in Table 3.

## 4. Decoding Beyond the Last Frozen Bit

Fazeli et al. displayed a combination algorithm between SCL with $L=2^{MF}$ and the nearest coset decoding in [6], which coincided with ML performance. Herein, we propose a new decoding hard-decision rule after the last frozen bit in BMS channels, which can also obtain close to ML performance.

**Definition 3**  (Polar Coset)**.** *Let C be a polar code with length $n=2^{m}$, given a binary information sequence $u_{0}^{i-1}$, where $i\in \left\{0,1,\cdots ,n-1\right\}$; we denote $C_{n}^{\left(i\right)}\left(u_{0}^{i-1}\right)$ as a polar coset:*

(13) $$C_{n}^{\left(i\right)}\left(u_{0}^{i-1}\right)=\sum_{j=0}^{i-1}u_{j}G_{2}^{⊗m}\left[j\right]+span\left\{G_{2}^{⊗m}\left[i:n-1\right]\right\},$$

*and polar coset space is spanned by all rows of $G_{2}^{⊗m}$ if i=0. Denote $C_{n}\left(u_{0}^{i-1}|u_{i}=0\right)$, $C_{n}\left(u_{0}^{i-1}|u_{i}=1\right)$ as zero-coset and one-coset, respectively,*

(14) $$C_{n}\left(u_{0}^{i-1}|u_{i}=0\right)=\sum_{j=0}^{i-1}u_{j}G_{2}^{⊗m}\left[j\right]+span\left\{G_{2}^{⊗m}\left[i+1:n-1\right]\right\},$$

(15) $$C_{n}\left(u_{0}^{i-1}|u_{i}=1\right)=G_{2}^{⊗m}\left[i\right]+C_{n}\left(u_{0}^{i-1}|u_{i}=0\right),$$

*where $G_{2}^{⊗m}\left[i\right]$ represents the (i+1)-th row of $G_{2}^{⊗m}$, and $G_{2}^{⊗m}\left[i:j\right]$ represents the (i+1)-th row to the (j+1)-th row of $G_{2}^{⊗m}$.*

According to the definition in [1], identity permutation is denoted by $\pi_{0}$. For symmetric channels *W*, there exists a permutation $\pi_{1}$ on output alphabet Y such that $\pi_{1}=\pi_{1}^{-1}$ and $W\left(y|1\right)=W(\pi_{1}\left(y\right)|0)$ for y∈Y. Let $x_{0}^{n-1}⊙y_{0}^{n-1}=\left\{x_{0}⊙y_{0},x_{1}⊙y_{1},\cdots ,x_{n-1}⊙y_{n-1}\right\}$, where $x_{i}⊙y_{i}$ is $\pi_{x_{i}}\left(y_{i}\right)$.

**Proposition 1.**  *Over BMS W, $y_{0}^{n-1}$ and ${\hat{u}}_{0}^{i-1}$ are, respectively, the received vector and decoded vector in SC decoding, then the transition probabilities of subchannels $W_{n}^{\left(i\right)}$ are*

(16) $$W_{n}^{\left(i\right)}\left(y_{0}^{n-1},{\hat{u}}_{0}^{i-1}|u_{i}=q\right)=W_{n}^{\left(i\right)}\left({\tilde{y}}_{0}^{n-1},0_{0}^{i-1}|u_{i}=q\right),$$

*where $q\in \left\{0,1\right\}$, $i\in \left\{0,1,\cdots ,n-1\right\}$, ${\tilde{y}}_{0}^{n-1}={\hat{u}}_{0}^{i-1}G_{2}^{⊗m}\left[0:i-1\right]⊙y_{0}^{n-1}$.*

**Proof.**  Applying symmetric property from Proposition 13 in [1], for any $a_{0}^{n-1}\in {\left\{0,1\right\}}^{n}$, we have

(17) $$W_{n}^{\left(i\right)}\left(y_{0}^{n-1},{\hat{u}}_{0}^{i-1}|u_{i}\right)=W_{n}^{\left(i\right)}\left(a_{0}^{n-1}G_{2}^{⊗m}⊙y_{0}^{n-1},{\hat{u}}_{0}^{i-1}⊕a_{0}^{i-1}|u_{i}⊕a_{i}\right)$$

Let $a_{0}^{i-1}={\hat{u}}_{0}^{i-1}$, $a_{i}=0$, $a_{i+1}^{n-1}=0$, ${\tilde{y}}_{0}^{n-1}=a_{0}^{n-1}G_{2}^{⊗m}⊙y_{0}^{n-1}$, then ${\hat{u}}_{0}^{i-1}⊕a_{0}^{i-1}=0,\;{\tilde{y}}_{0}^{n-1}={\hat{u}}_{0}^{i-1}G_{2}^{⊗m}\left[0:i-1\right]⊙y_{0}^{n-1}$. Hence, $W_{n}^{\left(i\right)}\left(y_{0}^{n-1},{\hat{u}}_{0}^{i-1}|u_{i}=q\right)=W_{n}^{\left(i\right)}\left({\tilde{y}}_{0}^{n-1},0_{0}^{i-1}|u_{i}=q\right)$ when $q\in \left\{0,1\right\}$. □

Due to the transition probabilities relationship of subchannels and synthetic channels in polarization process

(18) $$W_{n}^{\left(i\right)}\left(y_{0}^{n-1},u_{0}^{i-1}|u_{i}\right)=\sum_{u_{i+1}^{n-1}\in {\left\{0,1\right\}}^{n-i-1}}\frac{1}{2^{n-1}}W_{n}\left(y_{0}^{n-1}|u_{0}^{n-1}G_{2}^{⊗m}\right),$$

we change the condition of (1) into (19) from Proposition 1 and (18)

(19) $$\begin{array}{cc} \frac{W_{n}^{\left(i\right)}\left(y_{0}^{n-1},{\hat{u}}_{0}^{i-1}|u_{i}=0\right)}{W_{n}^{\left(i\right)}\left(y_{0}^{n-1},{\hat{u}}_{0}^{i-1}|u_{i}=1\right)} & =\frac{W_{n}^{\left(i\right)}\left({\tilde{y}}_{0}^{n-1},0_{0}^{i-1}|u_{i}=0\right)}{W_{n}^{\left(i\right)}\left({\tilde{y}}_{0}^{n-1},0_{0}^{i-1}|u_{i}=1\right)}\\ & =\frac{\sum_{x_{0}^{n-1}\in C_{n}\left(0_{0}^{i-1}|u_{i}=0\right)}W_{n}\left({\tilde{y}}_{0}^{n-1}|x_{0}^{n-1}\right)}{\sum_{x_{0}^{n-1}\in C_{n}\left(0_{0}^{i-1}|u_{i}=1\right)}W_{n}\left({\tilde{y}}_{0}^{n-1}|x_{0}^{n-1}\right)}. \end{array}$$

By switching channel transition probabilities into Hamming distance, the hard-decision rule after the last frozen bit under SC decoding is replaced to achieve ML bound in [6]. Therefore, we switch the origin rule of polar codes into finding minimum Hamming distance of two cosets $C_{n}\left(0_{0}^{i-1}|u_{i}=0\right),C_{n}\left(0_{0}^{i-1}|u_{i}=1\right)$ with ${\tilde{y}}_{0}^{n-1}$, that is to say

(20) $$\frac{\sum_{x_{0}^{n-1}\in C_{n}\left(0_{0}^{i-1}|u_{i}=0\right)}W_{n}\left({\tilde{y}}_{0}^{n-1}|x_{0}^{n-1}\right)}{\sum_{x_{0}^{n-1}\in C_{n}\left(0_{0}^{i-1}|u_{i}=1\right)}W_{n}\left({\tilde{y}}_{0}^{n-1}|x_{0}^{n-1}\right)}\Leftrightarrow \frac{\min_{x_{0}^{n-1}\in C_{n}\left(0_{0}^{i-1}|u_{i}=0\right)}d({\tilde{y}}_{0}^{n-1},x_{0}^{n-1})}{\min_{x_{0}^{n-1}\in C_{n}\left(0_{0}^{i-1}|u_{i}=1\right)}d({\tilde{y}}_{0}^{n-1},x_{0}^{n-1})}.$$

Under SCL decoding, aiming to ensure close to ML algorithm performance, we reserve all possible paths with $L=2^{MF}$ before the last frozen bit of polar codes, following employing (20) to compare the distance of two cosets to decode the rest of continued information bits.

In Section 3, we completely show the upper bound of mixing factor of polar codes with $R<\frac{1}{2}$ and achievable RM codes with $R=\frac{1}{2}$; so, herein, consider the situation in $R<\frac{1}{2}$, that is, $\tau \left(C\right)\geq \frac{n}{2}$. By the structure observation of $G_{2}^{⊗m}={\left[\begin{array}{cc} 1 & 0\\ 1 & 1 \end{array}\right]}^{⊗m}$ and subcode duality $C_{n}^{\left(n-i\right)}\left(0_{0}^{n-i-1}\right)={\left[C_{n}^{\left(i\right)}\left(0_{0}^{i-1}\right)\right]}^{⊥}\left(\frac{n}{2}\leq i\leq n-1\right)$ of Theorem 8 in [17], we induce expression of polar coset $C_{n}^{\left(i\right)}\left(0_{0}^{i-1}\right)$

(21) $$C_{n}^{\left(i\right)}\left(0_{0}^{i-1}\right)=\left\{\begin{array}{cc} {\left[C_{n}^{\left(n-i\right)}\left(0_{0}^{n-i-1}\right)\right]}^{⊥}, & 0\leq i\leq \frac{n}{2}-1\\ \underset{u_{i}\in \left\{0,1\right\}}{⋃}\left\{\left(c,c\right)\left|c\in C_{\frac{n}{2}}^{\left(i-\frac{n}{2}+1\right)}\left(0_{0}^{i-\frac{n}{2}-1},u_{i-\frac{n}{2}}=u_{i}\right)\right.\right\}, & \frac{n}{2}\leq i\leq n-1 \end{array}\right.$$

where ⊥ represents the dual of code. Therefore, we can recursively calculate the polar coset $C_{n}^{\left(i\right)}\left(0_{0}^{i-1}\right)$ using (21) in certain cases so as to obtain decoding results after the last frozen bit from (20). In fact, we merely provide a simple decision idea for decoding beyond the last frozen bit. Unfortunately, we do not give an appropriate and practical algorithm to recursively calculate minimum Hamming distance.

## 5. Conclusions

In this paper, aiming to prove the conjecture in [8], we show the upper bound of the mixing factor of decreasing monomial codes with code length $n=2^{m}$ and code rate $R\leq \frac{1}{2}$, when *m* is an even number. Meanwhile, we certify that this bound is reachable: there are two situations that reach the largest value if t=1 and t=2, and there is only one case for t≥3. Further, we propose a new decoding hard-decision rule with respect to the Hamming distance between cosets and the given vector beyond the last frozen bit of polar codes over BMS channels, but we do not give an effective and complete algorithm to calculate minimun distance, which provides us with purpose and motivation to continue exploring this work.

## Figures and Tables

**Table 1:** Some of the largest mixing factor values of decreasing monomial code C with length $n=2^{m}$, m=2t.

*t*	$n=2^{2t}$	$\max\left\{\mathrm{MF}\left(C\right)\right\}$
1	4	0
2	16	2
3	64	18
4	256	98
5	1024	450
6	4096	1922
7	16,384	7936

**Table 2:** Row indices of $G_{2}^{⊗m}$ and corresponding monomials.

$i_{f}$	$\mathrm{bin}(i_{f})$	*f*	deg(f)
0	00⋯00	$x_{0}x_{1}...x_{2t-1}$	2t
1	00⋯01	$x_{1}x_{2}...x_{2t-1}$	2t−1
⋮	⋮	⋮	⋮
$2^{2t}-2^{t+1}$	$\underset{t-1}{\underset{︸}{11\cdots 11}}\underset{t+1}{\underset{︸}{00\cdots 00}}$	$x_{0}x_{1}...x_{t}$	t+1
$2^{2t}-2^{t+1}+1$	$\underset{t-1}{\underset{︸}{11\cdots 11}}\underset{t+1}{\underset{︸}{00\cdots 01}}$	$h=x_{1}x_{2}...x_{t}$	*t*
$2^{2t}-2^{t+1}+2$	$\underset{t-1}{\underset{︸}{11\cdots 11}}\underset{t+1}{\underset{︸}{00\cdots 10}}$	$h^{\prime }=x_{0}x_{2}...x_{t}$	≤t
⋮	⋮	⋮	
$2^{2t}-1$	$\underset{t-1}{\underset{︸}{11\cdots 11}}\underset{t+1}{\underset{︸}{11\cdots 11}}$	1	

**Table 3:** The MF values of polar codes C based on PW construction with length $n=2^{m}$, m=2t.

*t*	*n*	$\max\left\{\mathrm{MF}\left(C\right)\right\}$	$\mathrm{MF}\left(C\right)\;\mathrm{with}\;R=\frac{1}{4}$	$\mathrm{MF}\left(C\right)\;\mathrm{with}\;R=\frac{1}{2}$
1	4	0		0
2	16	2	1	2
3	64	18	9	17
4	256	98	37	73
5	1024	450	193	385

## Data Availability

The data are contained within the article.

## Abbreviations

The following abbreviations are used in this manuscript:

BMS	Binary-input Memoryless Symmetric
SCL	Successive Cancellation List
ML	Maximum Likelihood
MF	Mixing Factor
SC	Successive Cancellation
CRC	Cyclic Redundancy Check
PC	Parity Check
PAC	Polarization-adjusted Convolutional
BEC	Binary Erasure Channel
MAP	Maximum A Posteriori
BI-AWGN	Binary-input Additive White Gaussian Noise
RM	Reed-Muller
WEF	Weight Enumeration Function
DE	Density Evolution
GA	Gaussian Approximation
PW	Polarization Weight
BP	Belief Propagation
SCS	Successive Cancellation Stack
